# Candidate Metabolomic Biomarkers of the Geographical-Indication Pear Gaoping Dahuangli (*Pyrus bretschneideri* Rehd.): An Exploratory Widely Targeted Metabolomics Study

**DOI:** 10.3390/foods15193457

**Published:** 2026-09-27

**Authors:** Ke Li, Huilan Wang, Ting Cao, Guozhen Wang, Yanjun Cheng, Yuqin Song, Xinxin Feng, Liulin Li

**Affiliations:** 1 College of Horticulture, Shanxi Agricultural University, Jinzhong 030801, China; 1708762@163.com (K.L.); 18534979020@163.com (T.C.); songyuqin@sxau.edu.cn (Y.S.); 2 Infrastructure Division, Shanxi Normal University, Taiyuan 030031, China; 3 Shanxi Key Laboratory of Food and Drug Safety Prevention and Control, Taiyuan 030032, China; whl0351@126.com (H.W.); zhen1718@163.com (G.W.); sjzxnjs@163.com (Y.C.); 4 Shanxi Inspection and Testing Center (Shanxi Institute of Standards and Metrology), Taiyuan 030032, China

**Keywords:** Gaoping Dahuangli, geographical indication, widely targeted metabolomics, flavonoids, terpenoids, biomarker, cultivar authentication

## Abstract

Gaoping Dahuangli is a landrace pear grown for more than four centuries in Gaoping County, Shanxi Province, and a National Geographical Indication (GI) product since 2013. What distinguishes it chemically from other pears—and how that distinction can be proved in the marketplace—has not been established. We profiled the fruit metabolome of Dahuangli and four commercial reference cultivars (Korla xiangli, Jingbaili, Clapp’s Favorite, and Akizuki) by widely targeted UHPLC-MS/MS metabolomics (five varieties × three biological replicates, single harvest season) and measured eight quality attributes. Of 2335 annotated metabolic features, 136 were consistently more abundant in Dahuangli than in every reference variety. Flavonoids (48) and terpenoids (25) made up over half of this set; the single most enriched compound was sophorabioside, an isoflavone, at a minimum fold change of 120.7×. Dahuangli combined the firmest flesh (7.27 kg/cm^2^) with the lowest sugar/acid ratio (38.6) and the yellowest peel (b* = 51.41). On this basis we propose a candidate two-tier authentication framework: 32 candidate biomarkers enriched more than 10-fold for laboratory confirmation, 104 further metabolites for corroborative pattern matching, and two field-measurable proxies—firmness and the sugar/acid ratio—for low-cost first-stage screening. These exploratory results give a four-century-old landrace its first objective chemical definition and offer regulators, producers, and consumers a candidate toolkit—two field-measurable proxies backed by a 32-compound laboratory panel—for exposing substitution and protecting the Dahuangli geographical indication, pending multi-year and multi-orchard validation.

## 1. Introduction

Gaoping Dahuangli (*Pyrus bretschneideri* Rehd.) [1,2] has been cultivated in Gaoping County, Shanxi Province, for more than 400 years and was designated a National Geographical Indication (GI) product in 2013 [3,4]. Its yellow-green peel, unusually firm flesh, and tart-sweet flavor give the fruit excellent storage and processing quality—and make it worth imitating. GI enforcement, however, still rests on origin paperwork rather than on any property of the fruit itself: there is no objective chemical test that distinguishes authentic Dahuangli from the cheaper commercial cultivars sold under its name [5,6].

Widely targeted metabolomics (WTM), which couples UHPLC-MS/MS with large compound libraries, can quantify hundreds to thousands of fruit metabolites in a single run and has been used to authenticate apple, grape, and citrus products [7,8,9,10,11]. In pear, WTM has separated *P. bretschneideri*, *P. ussuriensis*, and *P. pyrifolia* at the species level on the basis of carbohydrate, polyphenol, and amino acid profiles [12,13], and has compared pulp chemistry among Korla xiangli types [14]. The question that matters for GI enforcement is narrower and, to our knowledge, unanswered: can one landrace cultivar be told apart from the commercial varieties that substitute for it?

We addressed this question by comparing Dahuangli with four widely grown cultivars—Korla xiangli (*P. sinkiangensis*), Jingbaili (*P. ussuriensis*), Clapp’s Favorite (*P. communis*), and Akizuki (*P. pyrifolia*). The choice was deliberate. Substitution in the marketplace uses whatever sells cheaply, regardless of botanical species, and these four cultivars span the principal cultivated pear species in China; a metabolite that remains enriched against all four backgrounds simultaneously is unlikely to be an artifact of any single comparison. The design has a cost, which we state plainly: because the references are different species, cross-species divergence and cultivar specificity are confounded in the resulting panel. We therefore treat the biomarkers reported here as candidates and define, in Section 4.4, the intra-specific validation required to settle their status.

Specifically, we (i) profiled the fruit metabolome of Dahuangli and the four references by UHPLC-MS/MS-based WTM; (ii) extracted the metabolites consistently enriched in Dahuangli across all four backgrounds and tested their discriminatory power; (iii) asked how far this signature accounts for the cultivar’s unusual firmness, acidity, and peel color; and (iv) assembled the results into a tiered authentication framework compatible with field screening.

## 2. Materials and Methods

### 2.1. Plant Materials and Sample Collection

Fruits of Gaoping Dahuangli (*P. bretschneideri* Rehd.) and the four reference varieties were harvested at commercial maturity in 2024: Dahuangli from Gaoping County, Shanxi Province (35°48′ N, 112°55′ E), the core GI production area; Korla xiangli (*P. sinkiangensis* Yü) from Korla City, Xinjiang [15]; Jingbaili (*P. ussuriensis* Maxim.) from Beijing [16]; Clapp’s Favorite (*P. communis* L.) from Shandong Province [17]; and Akizuki (*P. pyrifolia* Nakai) from Sanmenxia City, Henan Province [18]. The four references represent the realistic substitution pool for GI infringement—high-volume commercial cultivars spanning the four principal cultivated pear species in China—so that any candidate marker must discriminate Dahuangli across maximally diverse genetic backgrounds.

For each variety, 30 fruits of uniform size and without visible defects were collected at random from 10 trees (3 fruits per tree) and transported to the laboratory within 24 h. For metabolomics, fruits were peeled with a stainless-steel peeler, removing the skin together with the outermost subepidermal flesh layer; the peeled flesh was cut into small pieces, pooled, frozen in liquid nitrogen, and stored at −80 °C; three biological replicates were prepared per variety, each consisting of pooled flesh from 10 fruits that were assigned to the pool at random from the full set of 30 fruits (i.e., pools mix fruits across trees rather than representing individual trees, and the pool is the biological experimental unit). Quality attributes were measured on individual fresh fruits (five per variety for fruit weight, firmness, soluble solids content, and skin color; three per variety for titratable acidity and soluble sugar). Quality and metabolomic measurements were performed on fruits drawn from the same batch and harvest; however, the two datasets were not paired at the individual-fruit level.

### 2.2. Quality Attribute Determination

Single-fruit weight was recorded on a digital balance (0.1 g). Firmness was measured with a GY-4 digital penetrometer (Bosean, Zhengzhou, China; 8-mm probe) and expressed as kg/cm^2^. Soluble solids content was read on a handheld digital refractometer (°Brix). Soluble sugar was determined by the anthrone method [19] and expressed as mg glucose equivalents per g fresh weight. Titratable acidity was determined by titration with 0.1 M NaOH to pH 8.2 and expressed as % malic acid equivalents. Peel color was measured with a CR-400 chromameter (Konica Minolta, Tokyo, Japan) at three equatorial positions per fruit, recording L*, a*, and b*. The sugar–acid ratio was calculated on a common mass basis as soluble sugar content (mg/g fresh weight, divided by 10 to express as % fresh weight) divided by titratable acidity (% fresh weight); it is therefore a soluble-sugar/titratable-acidity ratio, distinct from the more commonly used soluble-solids/acidity ratio.

### 2.3. Widely Targeted Metabolomics Analysis

#### 2.3.1. Metabolite Extraction

Frozen fruit powder (100 mg) was extracted with 1.0 mL methanol:water (3:1, *v*/*v*) containing 2-chlorophenylalanine (1 mg/L; purity 98%; J&K Scientific, Beijing, China; CAS 103616-89-3) as the internal standard. The internal standard was used solely to monitor the stability of the analytical run and was not used for quantification; all metabolite abundances are reported as peak areas. After vortexing and centrifugation (12,000× *g*, 10 min, 4 °C), the supernatant was filtered through a 0.22 μm PTFE membrane for UHPLC-MS/MS analysis.

#### 2.3.2. UHPLC-MS/MS Conditions

Chromatographic separation was performed on an ExionLC™ AD ultra-high-performance liquid chromatography system coupled to a SCIEX QTRAP 6500 triple-quadrupole mass spectrometer (SCIEX, Framingham, MA, USA) equipped with an electrospray ionization (ESI) source, operated in scheduled multiple reaction monitoring (MRM) mode with positive/negative polarity switching. The mass axis was calibrated with the manufacturer’s tuning solution (SCIEX). The analytical column was an Agilent SB-C18 (2.1 × 100 mm, 1.8 μm; Agilent Technologies, Santa Clara, CA, USA). Mobile phase A was ultrapure water containing 0.1% formic acid, and mobile phase B was acetonitrile containing 0.1% formic acid. The gradient program was: 0.00 min, 5% B; linear increase to 95% B at 9.00 min; 95% B held for 1 min; 10.00–11.10 min, decrease to 5% B; 5% B held for equilibration until 14 min. The flow rate was 0.35 mL/min, the column temperature was 40 °C, and the injection volume was 2 μL. The ESI source temperature was 500 °C; the ion-spray voltage was 5500 V in positive mode and −4500 V in negative mode; ion source gas I, gas II, and curtain gas were set to 50, 60, and 25 psi, respectively; the collision-activated dissociation parameter was set to high, and the collision gas (nitrogen) was set to medium. The declustering potential (DP) and collision energy (CE) were optimized for each MRM transition; according to the metabolites eluting within each time window, a specific set of MRM transitions was monitored in each period.

#### 2.3.3. Metabolite Identification and Quantification

Metabolite identification was performed using the MetWare database (MWDB) containing over 3000 annotated plant metabolites. Compound annotation criteria, appropriate for unit-resolution MRM acquisition, were: (1) retention time matching within ±0.1 min against the library entry; (2) co-detection of the characteristic quantifier and qualifier MRM transitions (compound-specific optimized declustering potential and collision energy) at the expected ion ratio; and (3) MS/MS fragment pattern matching against the library spectrum. Quantification was performed by MRM; because no compound-specific calibration curves were used, the resulting data are semi-quantitative (relative quantification based on peak areas, with the internal standard used only for run-stability monitoring). Peak integration and quality control were conducted using MultiQuant software v3.0 (SCIEX). The 2335 detected entries are annotated metabolic features from the scheduled-MRM library after merging of adducts and duplicate ions against the same library entry; features detected in both ionization modes are counted once, in the mode used for quantification; positional isomers that share transitions and co-elute are counted as a single entry; and features without structural annotation (unknowns) are not included in this count. Annotations are reported at three platform confidence levels (level 1: full MS/MS spectrum and retention-time match against the library, score ≥ 0.7; level 2: 0.5 ≤ score < 0.7; level 3: Q1/Q3/RT/DP/CE consistency); because no authentic-standard co-injection was performed in this study, all assignments correspond to putative identifications in Metabolomics Standards Initiative (MSI) terminology—i.e., platform confidence level 1 corresponds to MSI level 2, and platform levels 2–3 to MSI level 3; no assignment in this study reaches MSI level 1. Across the 1863 significantly differential metabolites, 674 were annotated at platform level 1, 436 at level 2, and 753 at level 3; within the 136-member core set, 47 are level 1; and 10 of the 32 Tier I candidate biomarkers are level 1 (Tables S1 and S2). Supplementary Tables S1–S6 accompany this article; the Q1/Q3 transitions, ionization modes, and identification confidence levels of the 32 Tier I candidate markers are collected in Table S4.

### 2.4. Quality Control and Data Preprocessing

QC samples were pooled from equal aliquots of all 15 samples, and four QC injections were interspersed through the analytical batch [20]. Technical reproducibility was assessed by Pearson correlation among QC replicates (r = 0.93–0.97) and by the coefficient of variation (CV) of peak areas across QCs (median CV = 19.4%; 74.4% of quantified features with CV < 30%); no features were excluded on the basis of QC variation, and no QC-based signal correction was applied because QC drift was negligible (Figure S1A). No total-ion-current normalization was applied; all analyses used peak areas directly, with log_2_ transformation only for OPLS-DA. Features not detected in a given variety (below the instrumental detection limit) are recorded by the platform as a uniform low-abundance baseline value, identical across replicates; fold changes computed against such baselines therefore represent conservative lower-bound estimates rather than exact ratios. Remaining sporadic missing values were imputed by k-nearest neighbors (R package impute; version 1.76.0). Data were unit-variance scaled for PCA, hierarchical clustering, and heatmaps; for OPLS-DA they were log_2_-transformed and mean-centered.

### 2.5. Statistical Analysis

PCA was computed with prcomp in R (version 4.3.2) on unit-variance-scaled data. OPLS-DA was performed with the R package MetaboAnalystR (version 4.0) [21] on log_2_-transformed, mean-centered data, and models were validated by 200-permutation testing [22]. A metabolite was counted as significantly differential (SDM) in a pairwise comparison when it met both of two criteria: variable importance in projection (VIP) > 1.0 and |log_2_ fold change| ≥ 1.0. Venn analysis identified SDMs shared across the four comparisons of Dahuangli with each reference variety. Hierarchical clustering of scaled intensities was visualized with ComplexHeatmap. In addition to the multivariate criteria, univariate Welch’s *t*-tests were computed for each metabolite in each pairwise comparison, and *p*-values were adjusted across all 2335 detected features using the Benjamini–Hochberg (BH) procedure (adjusted *p*-values are reported in Table S5); of the 136 core metabolites, 99 also pass BH-adjusted *p* < 0.05 in all four comparisons. Chemical-class enrichment (Section 3.3) was tested with a one-sided Fisher’s exact test against the annotated background, with BH correction across classes.

Quality attributes were compared among varieties by one-way ANOVA with Tukey’s HSD post-hoc test at *p* < 0.05. Correlations between quality attributes and metabolite classes were computed on variety means (*n* = 5), because fruit-level pairing between the two datasets was not recorded (Section 2.1); significance was assessed by an exact two-sided permutation test over all 120 possible pairings of varieties; no multiple-testing correction was applied across the 48 trait–class pairs, and the resulting *p*-values are reported as exploratory. Class abundances were defined as summed peak areas of the 136 Dahuangli-enriched metabolites (Section 3.2) within each of six major classes (amino acids and derivatives, flavonoids, lignans and coumarins, organic acids, phenolic acids, terpenoids).

## 3. Results

### 3.1. Global Metabolome Overview and Data Reliability

Four QC injections interspersed through the batch gave Pearson correlations of r = 0.93–0.97 (Figure S1) and a median peak-area CV of 19.4% (74.4% of features below 30%), so instrument drift is small relative to biological signal. A PCA of all 19 samples (15 biological, 4 QC) makes the same point visually: the QCs sit in a tight cluster while the five varieties spread apart along PC1 (25.89%) and PC2 (21.74%) (Figure 1A).

In total, 2335 annotated metabolic features spanning 13 chemical classes were detected (Figure 1B). Terpenoids (14.73%), flavonoids (14.22%), and phenolic acids (11.73%) were the largest annotated classes, ahead of amino acids and derivatives (10.06%) and alkaloids (9.16%); 16.87% of features fell outside specific annotation (“Others”). This composition is typical of pear flesh chemistry, in which phenylpropanoid and isoprenoid pathways carry much of the quality-related variation [23].

### 3.2. Identification of the Core Dahuangli-Specific Metabolome

Each reference cultivar was first compared with Dahuangli by OPLS-DA. All four models performed well (R^2^Y = 1.000, Q^2^ = 0.985–0.993 across the four models; 200-permutation test *p* < 0.005; Figures S2 and S3), and the dual criterion of VIP > 1.0 plus |log_2_FC| ≥ 1.0 gave 991 SDMs against Korla xiangli, 1056 against Jingbaili, 1075 against Clapp’s Favorite, and 1219 against Akizuki (Figure 2A), corresponding to 1863 unique SDMs in the union of the four comparisons. Counts this large are expected between cultivars of different species and are of no direct use for authentication.

Two further filters turned them into a usable signature. The first was direction. An authentication marker must be something Dahuangli has, not something it lacks—absence of a compound is easily produced by ripeness, storage, or processing and proves nothing about identity. We therefore kept only the up-regulated side of each comparison: 695 metabolites more abundant in Dahuangli than in Jingbaili, 614 than in Korla xiangli, 487 than in Clapp’s Favorite, and 844 than in Akizuki (Figure 2A, up-regulated bars). Even after this restriction, each comparison alone still carried several hundred candidates, including dozens to hundreds found in no other comparison (unique sets of 53–216; Figure 2B), so no single pairwise result could serve as the signature.

The second filter was consistency across references: the intersection of the four up-regulated sets (Figure 2B). A metabolite survived only if it was more abundant in Dahuangli than in every reference variety at once. Exactly 136 metabolites met this condition (Figure 2B, center), and they constitute the candidate core Dahuangli signature examined below (Table S1).

### 3.3. Chemical Identity of the Dahuangli-Specific Metabolome

The 136 core metabolites are not evenly distributed across chemical classes. Flavonoids dominate (48 metabolites, 35.3%), followed by terpenoids (25, 18.4%) and lignans and coumarins (11, 8.1%); together these three classes account for 61.8% of the core set (Figure 3A). In the global metabolome the same classes make up only 34.1% (Figure 1B), and enrichment-factor analysis confirms that flavonoids are the only class significantly over-represented in the core set (2.48-fold, Fisher’s exact test *p* < 0.001; Figure 3B). Dahuangli’s specificity therefore lies not in novel chemistry but in concentrated enrichment within existing pathways.

At the subclass level (Figure 3C), flavones (17, 35.4% of flavonoids) and flavonols (15, 31.3%) predominate, with smaller contributions from anthocyanidins (4), flavanones (3), chalcones (3), and isoflavones (3). Within terpenoids, sesquiterpenoids (9, 36.0%) and triterpenes (8, 32.0%) are the largest groups. The co-enrichment of flavonoids—UV protectants and color determinants—and of terpenoids, many of which are cuticle and antimicrobial components, is consistent with adaptation to the high-radiation, arid conditions of the Loess Plateau [24].

### 3.4. Quantitative Specificity and Biomarker Hierarchy

The 136 core metabolites differ widely in how strongly they are enriched. For each metabolite we took the minimum fold change (min FC) across the four pairwise comparisons—the most conservative measure of specificity. The distribution is right-skewed (median 3.46×): most markers are enriched only a few-fold, while a small number show extreme specificity (Figure 4A). Sophorabioside, tentatively annotated as an isoflavone glycoside, is the only compound above 100-fold (min FC = 120.7×); it is followed by chrysoeriol-7-O-gentiobioside (99.8×), peonidin-3-O-(6″-O-caffeoyl)glucoside (94.4×), and apigenin-7-O-(6″-p-coumaryl)glucoside (87.8×)—all four tentatively annotated at the platform confidence levels given in Table S2 and, to our knowledge, not previously reported in *P. bretschneideri.* A heat map of the same markers shows that the enrichment is consistent across the three Dahuangli replicates and absent from all four reference varieties (Figure 4B).

This quantitative spectrum motivates a two-tier biomarker hierarchy (Table S2). Tier I comprises the 32 metabolites with min FC > 10× and is intended for confirmatory identification; it is dominated by flavonoids (20, 62.5%) and terpenoids (6, 18.8%), with two phenolic acids and one representative each of amino acids and derivatives, lignans and coumarins, organic acids, and other classes (Figure 4A). Tier II comprises the remaining 104 metabolites (min FC 2.0–9.4×) and serves for corroborative pattern matching in samples that deviate through natural metabolic variation.

### 3.5. Metabolic Basis of Dahuangli’s Distinctive Quality Attributes

Dahuangli’s phenotype is unusual in three directions at once (Figure S4; Table S3). Its flesh is the firmest of the five varieties (7.27 kg/cm^2^, 49.4% above the five-variety mean of 4.87; *p* < 0.05). It carries the least soluble sugar (73.40 mg/g) together with the second-highest titratable acidity (0.190%, after Jingbaili’s 0.227%), giving a sugar–acid ratio of 38.6—nowhere near the other varieties (54.8–163.2). And its peel is the yellowest (b* = 51.41), with a slightly positive a* (3.92) between the green-shifted Korla xiangli (−5.52) and Clapp’s Favorite (−3.81) and the red-russet Akizuki (13.36).

Correlating the eight quality traits with the six major metabolite classes across the five variety means (exact permutation test over all 120 variety pairings) gave five exploratory associations (Figure 5); because the minimum achievable *p* at *n* = 5 is 1/120 ≈ 0.008 and no multiple-testing correction was applied across the 48 trait–class pairs, these associations are hypothesis-generating leads rather than confirmed relationships. Peel yellowness (b*) tracked four classes: amino acids and derivatives (*r* = 0.97, *p* = 0.008), terpenoids (*r* = 0.93, *p* = 0.042), organic acids (*r* = 0.92, *p* = 0.042), and lignans and coumarins (*r* = 0.91, *p* = 0.017). Firmness tracked phenolic acids (*r* = 0.90, *p* = 0.025). Sugar-related traits showed the expected negative trends against flavonoids but none reached significance. With only five independent variety means, these correlations are leads, not proof; their value is that they point in the same direction as the chemistry below.

Two chemical threads connect the signature to the phenotype. For firmness, the core set contains eight triterpenes—including quinovic acid-3-O-rhamnoside and 2,3-dihydroxyolean-12-en-28-oic acid—that are known cuticular-wax constituents able to stiffen the cutin matrix [23,25]. For color, the highly enriched acylated flavonoids (peonidin-3-O-(6″-O-caffeoyl)glucoside, 94.4×; apigenin-7-O-(6″-p-coumaryl)glucoside, 87.8×) absorb at longer wavelengths than their non-acylated counterparts and are associated with stable yellow hues [26]; this is consistent with the class-level flavonoid–b* correlation in Figure 5, and we present this mechanism as a hypothesis.

### 3.6. Authentication Framework and Validation

A PCA built on only the top 20 biomarkers (ranked by mean VIP across the four OPLS-DA models) separates all five varieties cleanly, with PC1 and PC2 explaining 74.2% and 13.2% of the variance (Figure 6A). Dahuangli sits at PC1 ≈ +7.3 against ≤ −0.1 for every reference variety, and its three replicates cluster tightly. A 20-compound assay therefore retains essentially all of the discriminatory information in the full panel.

Metabolite assays are impractical outside the laboratory, so as a proof of concept we looked for physical proxies. Two are discriminatory in this dataset: firmness > 6.5 kg/cm^2^ and a sugar–acid ratio < 45 jointly separate Dahuangli from all four references (Figure 6B). At the individual-fruit level (three fruits per variety), no overlap was observed in this dataset between Dahuangli (36.8–40.8) and the nearest reference, Clapp’s Favorite (52.1–56.7), whereas firmness alone leaves a marginal overlap zone (one Dahuangli fruit at 6.32 kg/cm^2^ falls below the threshold and one Korla xiangli fruit at 6.45 kg/cm^2^ lies just below it), which is precisely why the protocol requires both proxies jointly, backed by LC-MS/MS confirmation. The joint rule nonetheless does not rescue every individual fruit: the 6.32 kg/cm^2^ Dahuangli fruit fails the firmness criterion regardless of its sugar–acid ratio, so the screen has a non-zero false-negative rate at the single-fruit level. Borderline samples—those failing one criterion narrowly while passing the other—should therefore be forwarded directly to LC-MS/MS confirmation rather than rejected. The thresholds are empirical midpoint classifiers fitted on this dataset (midpoints between the Dahuangli mean and the nearest reference mean; the resulting sugar–acid midpoint of 46.7 was rounded down to 45 as a slightly conservative integer threshold) and are dataset-specific. The full protocol (Figure 6C) is accordingly two-stage: Stage 1 screening by firmness and sugar/acid ratio; Stage 2 confirmation of flagged or disputed samples by LC-MS/MS against the 32 Tier I biomarkers, with the 104 Tier II metabolites available when a fuller pattern is needed. A portable implementation—handheld penetrometer for firmness, pocket refractometer for soluble solids, and portable titration for acidity, with the anthrone assay retained as the laboratory reference method—would allow the screen to be run outside the laboratory but remains to be validated in a dedicated field phase. Because the portable variant substitutes refractometric soluble solids for anthrone-measured soluble sugar, a soluble-solids/acidity threshold would require separate calibration and cannot simply reuse the <45 cut-off derived from anthrone data. The framework is a candidate one, pending the intra-specific and multi-origin validation set out in Section 4.4.

## 4. Discussion

### 4.1. The Distinctive Metabolic Identity of Gaoping Dahuangli

The metabolomic landscape of Gaoping Dahuangli reveals a cultivar that has carved out a unique chemical niche within the genus Pyrus. While the four reference varieties—Korla xiangli (*P. sinkiangensis*), Jingbaili (*P. ussuriensis*), Clapp’s Favorite (*P. communis*), and Akizuki (*P. pyrifolia*)—each possess characteristic metabolic profiles, none approach the extreme enrichment observed in Dahuangli relative to these four references. The identification of 136 consistently up-regulated metabolites, with 1 exceeding 100-fold enrichment and 32 exceeding 10-fold, suggests that Dahuangli’s metabolic distinctiveness is not merely quantitative but involves differences in secondary-metabolite profiles.

This observation resonates with emerging evidence that traditional landrace cultivars often retain metabolic traits lost in modern breeding programs [27]. The highest enrichment of sophorabioside (min FC = 120.7×)—an isoflavone glycoside—hints at the preservation of ancestral biosynthetic pathways or the emergence of novel enzymatic activities through centuries of local selection. The accumulation of glycosylated and acylated flavonoids in Dahuangli is consistent with—but does not demonstrate—altered glycosyltransferase activity or relaxed substrate specificity of existing enzymes; testing this warrants further investigation, while its candidate utility for authentication can be evaluated independently.

The dominance of flavonoids (35.3%) and terpenoids (18.4%) among the Dahuangli-enriched metabolites is particularly intriguing given their shared ecological functions. Both compound classes accumulate in plant cuticles and epidermal tissues, where they serve as UV protectants, antimicrobial agents, and structural components [28]. The Loess Plateau region, characterized by intense solar radiation, large diurnal temperature fluctuations, and limited precipitation, may have exerted selective pressure favoring cuticle-enriched chemotypes. This environmental filtering hypothesis aligns with the observation that Korla xiangli—cultivated in similarly arid Xinjiang conditions—shares some metabolic similarities with Dahuangli, though without the extreme enrichment of specific biomarkers.

### 4.2. Mechanistic Insights into Quality Attribute Formation

The exceptional fruit firmness of Dahuangli (7.27 kg/cm^2^) has long been recognized by growers and consumers, yet its biochemical basis remained obscure. Our metabolomic data suggest a candidate mechanistic explanation, which we present as a hypothesis: the accumulation of triterpenes (8 metabolites, 32.0% of terpenoids) and lignans and coumarins (11 metabolites, 8.1% of total). These pentacyclic triterpenes and phenylpropanoid oligomers are well-established constituents of fruit cuticular waxes and cell walls, where they modulate viscoelastic properties and mechanical resilience [23,25].

In cuticle ultrastructure, triterpenic acids intercalate with cutin polyester matrices and wax crystal domains, modulating viscoelastic properties and mechanical resilience [29]. The high triterpene content in Dahuangli likely enhances cuticle toughness, reducing fruit softening during ripening and postharvest storage. This interpretation is supported by comparative studies in sweet cherry, where cultivars with elevated triterpene wax content exhibit significantly slower firmness loss during cold storage [29]. The correlation between triterpene abundance and firmness in our dataset suggests that cuticle-associated triterpenes serve as metabolic proxies for storage quality, a finding with immediate applications for breeding and postharvest management.

The “low sugar–high acid” profile of Dahuangli—unique among the five varieties—reflects distinctive carbon partitioning at the metabolic network level. While modern fresh-eating cultivars (Jingbaili, Akizuki) have been selected for high soluble solids content [15,17], Dahuangli maintains elevated titratable acid (0.190%) with concomitantly reduced sugar accumulation (73.40 mg/g). This pattern is consistent with, but does not demonstrate, altered carbon partitioning—either reduced sugar accumulation or sustained acid retention during ripening; flux-level confirmation would require isotope-labelling experiments.

The sugar–acid ratio of 38.6 in Dahuangli stands in stark contrast to the 54.8–163.2 range observed in the reference varieties, positioning this cultivar in a unique sensory niche. From an evolutionary perspective, high acidity may confer microbial protection in the arid Loess Plateau environment, where fruit desiccation and pathogen pressure select for chemical defense mechanisms. From a culinary perspective, this profile explains Dahuangli’s traditional use in processed products (dried pear, vinegar, preserved fruit) where acidity enhances flavor complexity and microbial stability, rather than fresh consumption where sweetness dominates consumer preference.

The yellow coloration (b* = 51.41) emerges as another metabolically tractable trait. The high accumulation of acylated flavonoids—particularly peonidin-3-O-(6″-O-caffeoyl)glucoside (min FC = 94.4×) and apigenin-7-O-(6″-p-coumaryl)glucoside (min FC = 87.8×)—provides a mechanistic basis for this visual signature. Acylation with aromatic acids (caffeic, coumaric) shifts flavonoid absorption maxima toward longer wavelengths, producing yellow to orange hues that are more stable to pH variation and light exposure than non-acylated counterparts [26].

This biochemical specialization offers dual advantages: visual distinctiveness for consumer recognition and photoprotection for the underlying fruit tissues. The positive a* value (3.92) in Dahuangli—a shift in the red direction—further distinguishes it from the green-shifted Korla xiangli and Clapp’s Favorite (negative a*), suggesting that acylated flavones rather than chlorophyll degradation products dominate the color phenotype. The tight correlation between acylated flavonoid content and b* values across our sample set supports the use of simple colorimetric measurements as rapid, non-destructive proxies for metabolic authentication.

### 4.3. Comparative Context and Authentication Implications

When placed in the broader context of fruit metabolomics, Dahuangli’s metabolic profile reveals both convergent and divergent evolutionary patterns. The flavonoid-terpenoid co-enrichment observed here mirrors patterns in some wine grape cultivars, where anthocyanins and triterpenic acids co-accumulate in response to terroir conditions [30]. However, the magnitude of enrichment in Dahuangli (min FC up to 120.7×, with several compounds above 50×) has, to our knowledge, no precedent in the pear literature and suggests unique regulatory mutations rather than mere quantitative modulation.

Compared to previous pear authentication studies, our WTM approach offers several methodological advances. Zheng et al. [12] identified differential metabolites among *P. bretschneideri*, *P. ussuriensis*, and *P. pyrifolia* using WTM, selecting D-xylose, formononetin, procyanidin A1, and β-nicotinamide mononucleotide as key quality-associated markers. However, their study focused on species-level discrimination rather than cultivar-specific authentication within *P. bretschneideri*. Jiang et al. [14] applied WTM to compare metabolome and nutritional quality among three types of Korla fragrant pears with different appearances, revealing significant differences in primary metabolites and volatile compounds. Our study bridges these approaches by combining deep metabolite coverage (2335 compounds), quantitative rigor (MRM-based FC values), and multi-tier statistical validation (OPLS-DA, Venn analysis, PCA), enabling both candidate authentication and biological interpretation.

The proposed two-tier authentication framework addresses practical constraints that have limited the adoption of metabolomic methods in GI protection [4]. Tier I biomarkers (32 metabolites, min FC > 10×) provide a quantitative, reproducible basis for candidate authentication that can support regulatory enforcement and legal proceedings. Tier II pattern matching (104 metabolites) accommodates natural metabolic variation arising from seasonal, environmental, or preharvest factors, ensuring robustness across production years. The integration of simple quality parameters (firmness, sugar–acid ratio) provides a low-cost first-stage (Stage 1) screen that does not require sophisticated analytical equipment, with laboratory confirmation reserved for flagged samples.

This graded approach balances the diagnostic certainty required for fraud prosecution with the operational flexibility needed for routine quality control. For industry implementation, we envision a two-stage testing protocol: Stage 1, rapid physical/chemical screening (firmness + sugar/acid ratio), which is not a stand-alone proof of authenticity; and Stage 2, confirmatory LC-MS/MS analysis of Tier I biomarkers, required for any positive, flagged, or disputed sample.

### 4.4. Future Directions and Concluding Perspectives

Several avenues emerge from this work that merit sustained investigation. The structural elucidation of the highest-enrichment biomarkers—sophorabioside, chrysoeriol-7-O-gentiobioside, and peonidin-3-O-(6″-O-caffeoyl)glucoside—remains a priority; while our MS/MS data support their classifications, definitive structure assignment requires nuclear magnetic resonance (NMR) spectroscopy. Once confirmed, these compounds could serve as multi-marker diagnostics for rapid authentication kits.

Multi-omics profiling—incorporating stable isotope ratios (δ^13^C, δ^15^N, δD) and elemental fingerprints—would complement metabolomic data with geographic provenance markers, creating a comprehensive “chemical passport” for GI verification [31,32]. Such integration is particularly relevant given that metabolic profiles can be influenced by cultivation practices and seasonal variation, whereas isotopic signatures reflect underlying geology and climate.

Finally, sensory-metabolomic correlation studies would bridge the gap between chemical composition and consumer perception. While our data establish objective chemical differences, the question of which specific compounds contribute to Dahuangli’s distinctive flavor and mouthfeel remains open. Gas chromatography-olfactometry (GC-O) and electronic tongue systems could deconstruct the sensory experience into its chemical constituents, guiding quality-directed breeding and processing optimization.

Several limitations of this exploratory study should be noted. First, Dahuangli was sampled from its core GI production area in a single harvest year; because the GI designation is legally tied to that region, this sampling defines the authentication target, but within-region variation across orchards and years, Dahuangli grown outside the GI region, and same-species cultivars (other *P. bretschneideri* landraces such as ‘Dangshansu’ and ‘Lianglizaosu’) remain to be tested in a validation phase. Second, the metabolomic data are semi-quantitative (relative peak areas), and most identifications are library-match assignments; confirmation of the Tier I candidate markers with authentic standards and targeted quantification is required before operational use. Third, the two field-screening thresholds are empirical midpoints fitted on this five-variety dataset; both traits vary with season, orchard, and maturity, so the thresholds require recalibration across harvest years, and the firmness proxy in particular leaves a marginal overlap zone that the sugar–acid ratio and the LC-MS/MS tier are designed to close. Finally, metabolomic pools of ten fruits average out within-cultivar variation, and the effective replication is three pools per variety. In addition, the exact maximum storage duration of samples and extracts and the injection-order randomization scheme were not recorded in the analytical platform’s report, and are acknowledged here as unverifiable run-metadata gaps.

## 5. Conclusions

This exploratory study provides the first widely targeted metabolomic characterization of Gaoping Dahuangli pear, a National Geographical Indication product from Shanxi Province, China. We identified 136 metabolites consistently enriched in Dahuangli relative to four commercial substitute cultivars, with flavonoids (35.3%) and terpenoids (18.4%) dominating the enriched set. On this basis, a candidate two-tier authentication framework is proposed, comprising 32 high-specificity candidate biomarkers (min FC > 10×) and 104 supporting biomarkers (min FC 2–10×), together with two low-cost field-screening proxies. Quality assessment confirmed Dahuangli’s distinctive “high firmness–low sugar–high acid” profile, with firmness 49.4% above the five-variety average and a sugar–acid ratio of 38.6. These findings suggest hypotheses regarding the metabolic basis of Dahuangli’s quality attributes and provide a preliminary foundation for GI product authentication, variety improvement, and future functional studies; the framework is not yet ready for routine regulatory application: multi-year, multi-orchard validation—including same species *P. bretschneideri* cultivars—and authentic standard confirmation of the candidate markers are the essential next steps.

## Figures and Tables

**Figure 1:**
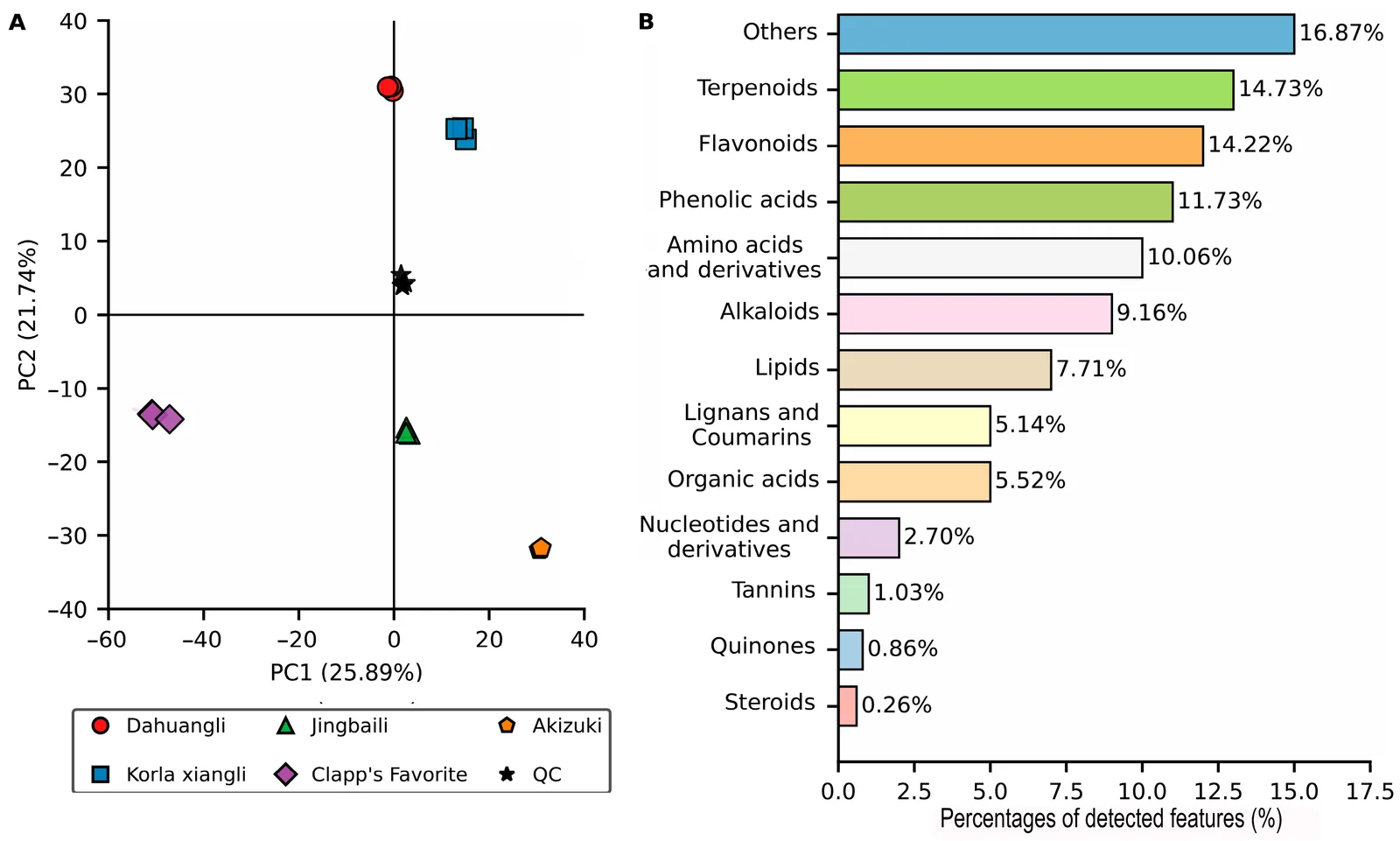
Global overview of the five-variety pear metabolome. (**A**) PCA score plot of all 19 samples (15 biological replicates, 4 quality-control (QC) injections) computed from unit-variance-scaled peak areas of 2335 annotated metabolic features; PC1 and PC2 explain 25.89% and 21.74% of the total variance. The tight QC cluster indicates negligible instrument drift relative to biological variation. (**B**) Percentage of detected annotated features belonging to each of the 13 chemical classes (feature counts rather than signal abundance).

**Figure 2:**
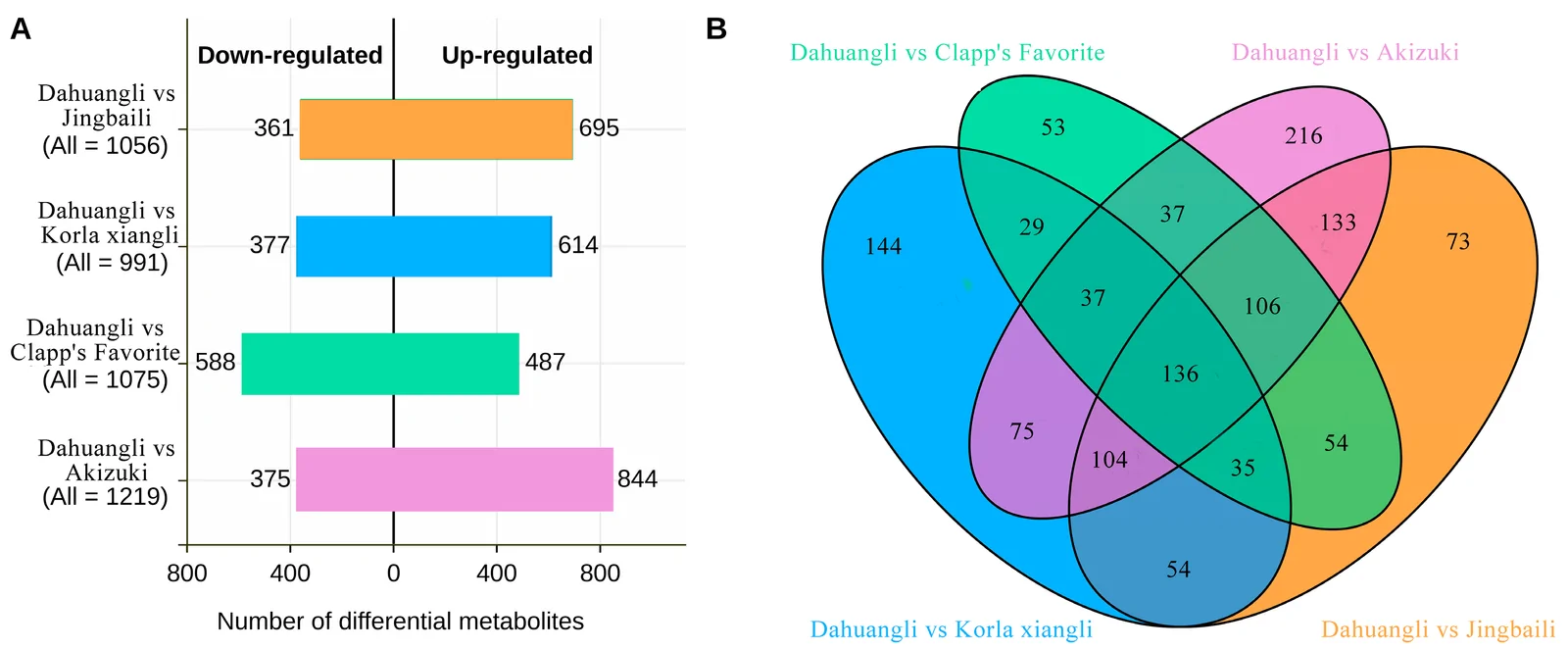
Definition of the core Dahuangli-enriched metabolite set. (**A**) Numbers of significantly differential metabolites (SDMs; VIP > 1.0 and |log_2_FC| ≥ 1.0) in each pairwise OPLS-DA comparison of Dahuangli against a reference cultivar, split by regulation direction; right-hand bars are metabolites more abundant in Dahuangli (up-regulated). (**B**) Four-way Venn diagram of the up-regulated sets. The central intersection—136 metabolites enriched in Dahuangli against every reference variety—defines the candidate core signature; figures in non-overlapping sectors are metabolites unique to a single comparison.

**Figure 3:**
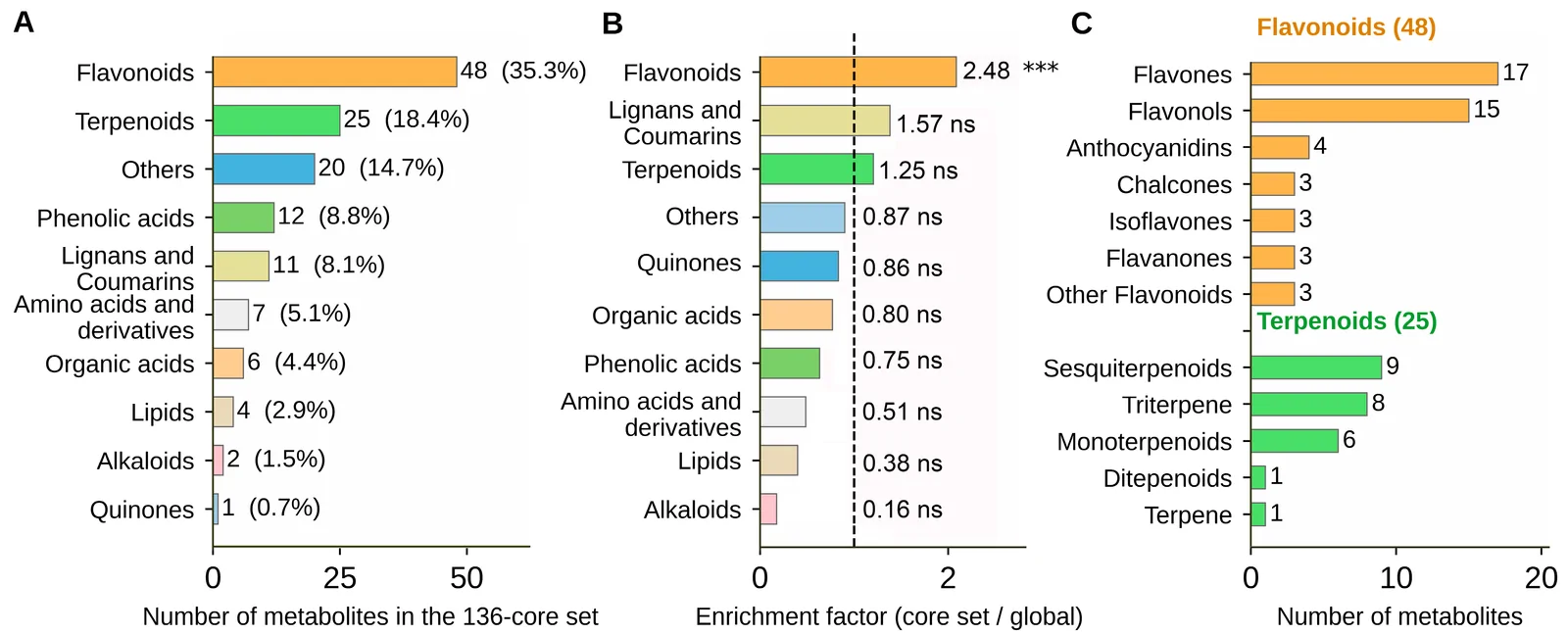
Chemical composition of the 136 core Dahuangli-enriched metabolites. (**A**) Class-level distribution; flavonoids (48), terpenoids (25), and lignans and coumarins (11) together account for 61.8% of the set. (**B**) Enrichment factor of each class in the core set relative to its share of the full background of all 2335 detected features (all classes, including “Others”, are computed against the same background); the dashed line marks 1 (no enrichment); *** *p* < 0.001, ns = not significant (Fisher’s exact test). (**C**) Subclass breakdown of the flavonoid (orange) and terpenoid (green) fractions.

**Figure 4:**
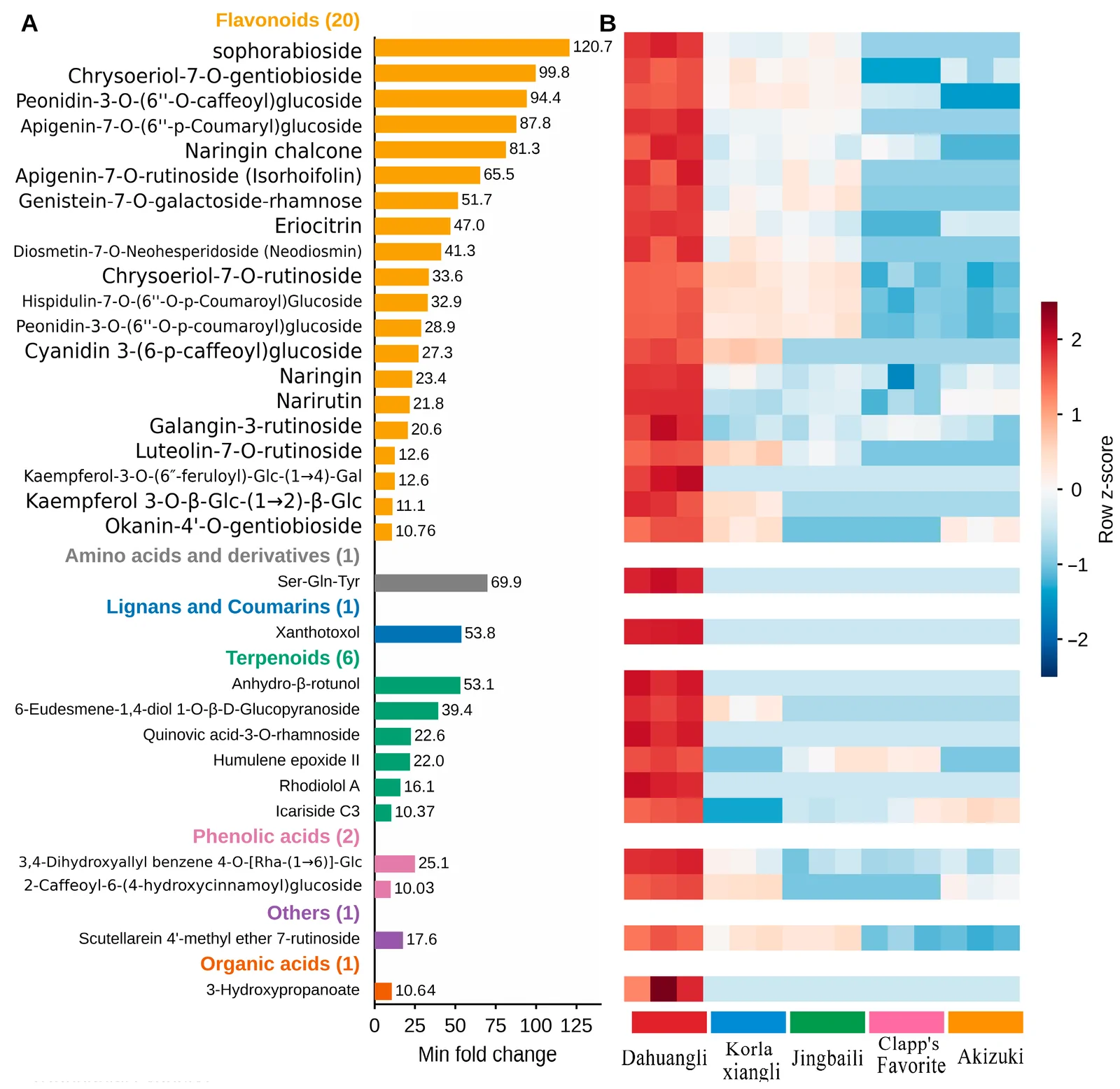
Quantitative specificity of the 32 Tier I biomarkers. (**A**) Minimum fold change (min FC—the smallest fold change across the four pairwise comparisons with Dahuangli) for each Tier I biomarker (min FC > 10×), grouped and colored by chemical class; bar labels give the min FC value (long systematic names are abbreviated for readability; full names are given in Table S2). The smallest min FC is 10.03×, above the Tier I threshold of 10×. (**B**) Heat map of row-scaled (z-score) peak areas of the same 32 biomarkers across all 15 samples, showing consistent enrichment in Dahuangli and low abundance in every reference cultivar.

**Figure 5:**
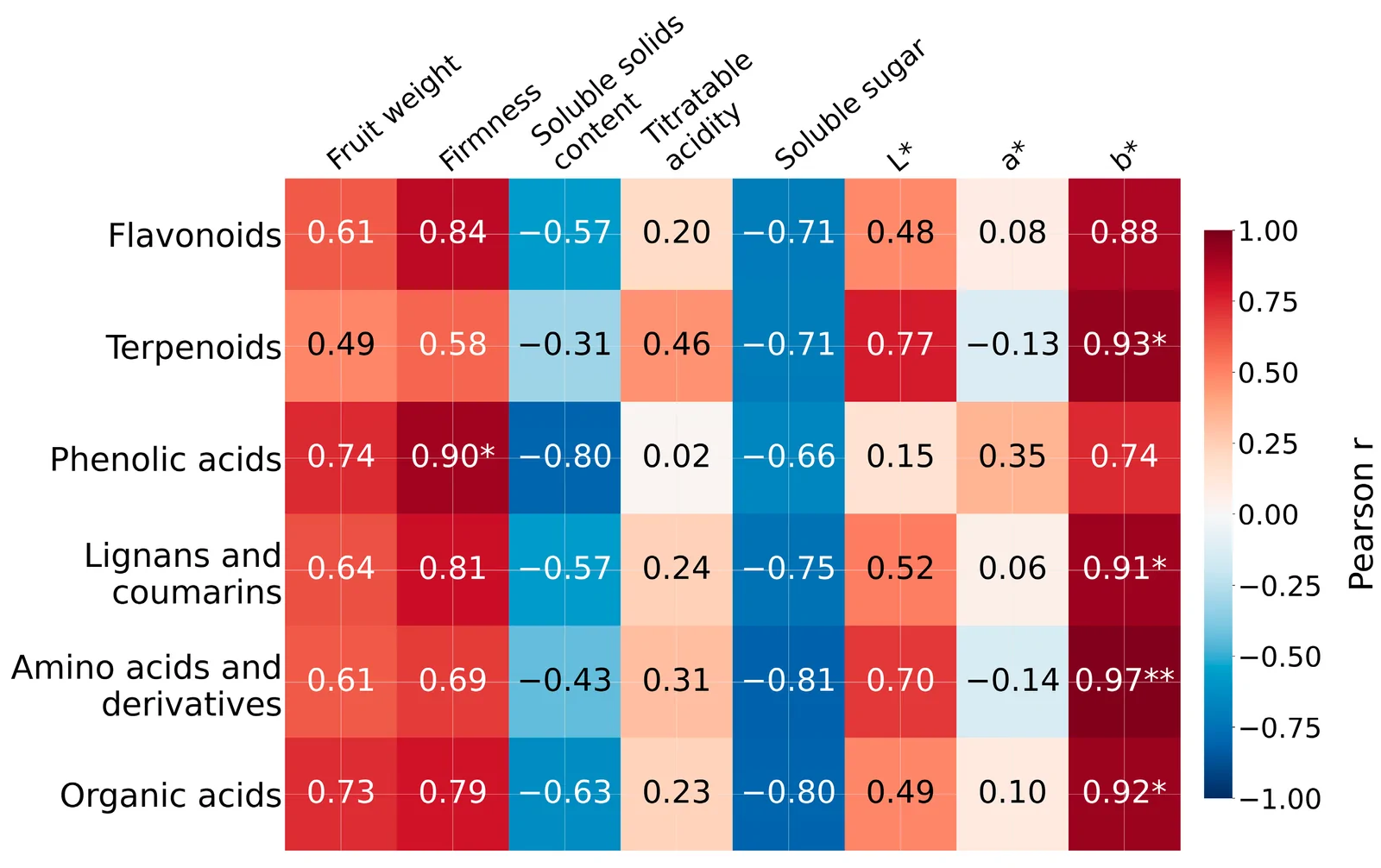
Pearson correlations between eight fruit quality traits and the summed peak areas of the 136 core metabolites within six major chemical classes, computed on variety means (*n* = 5). Significance was assessed by an exact two-sided permutation test over all 120 possible variety pairings: * *p* < 0.05, ** *p* < 0.01. *p*-values are exploratory (minimum achievable *p* = 1/120 at *n* = 5 variety means) and are not corrected for multiple testing.

**Figure 6:**
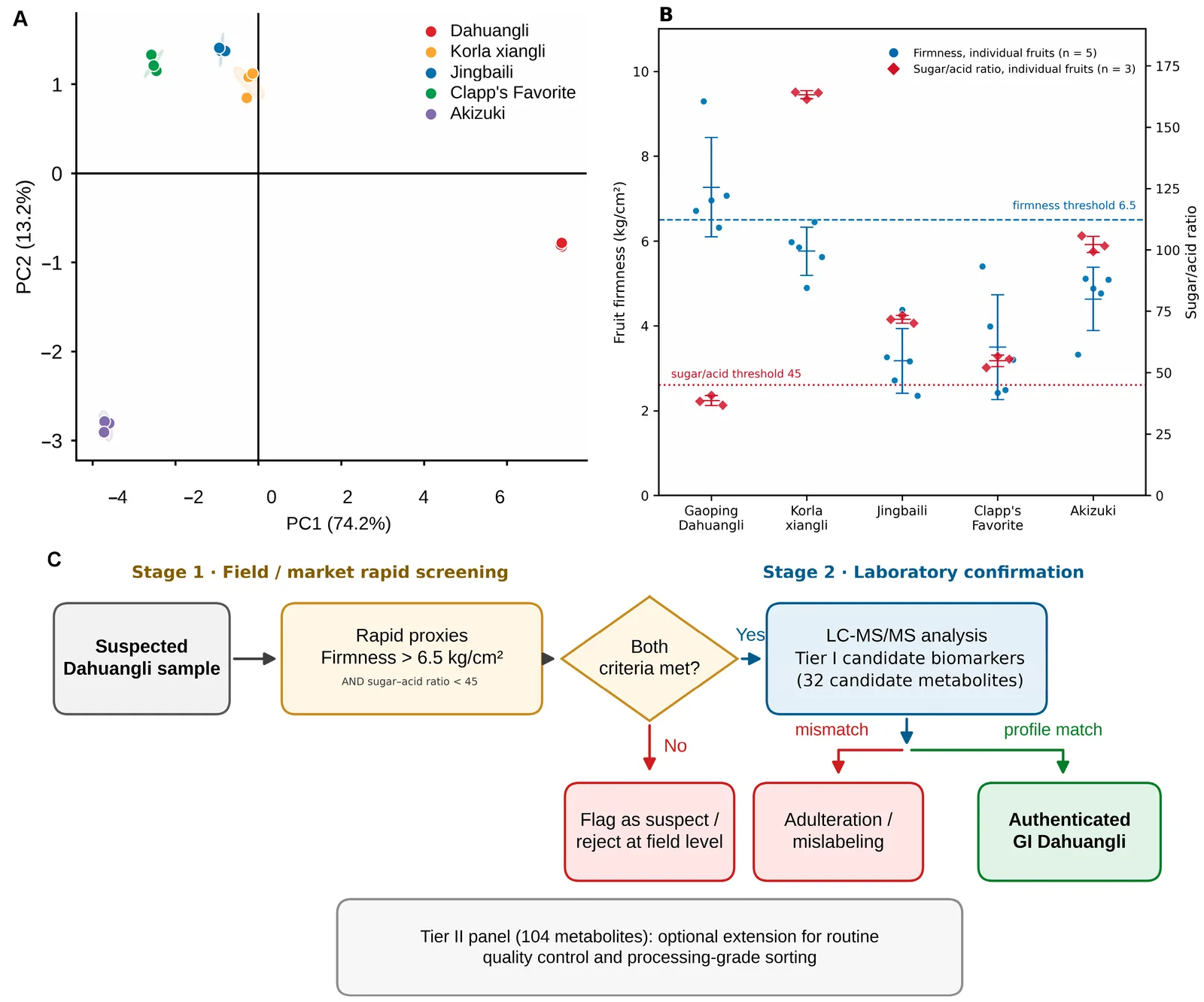
Biomarker-based authentication of Dahuangli. (**A**) PCA of the top 20 biomarkers (ranked by mean VIP across the four OPLS-DA models) separates all five varieties (PC1 = 74.2%, PC2 = 13.2% of variance). (**B**) Two field-measurable proxies—firmness > 6.5 kg/cm^2^ (left axis, *n* = 5 fruits per variety) and sugar–acid ratio < 45 (right axis, n = 3 individual fruits per variety; soluble sugar as % fresh weight divided by titratable acidity as %)—shown as individual values with mean ± SD; the sugar–acid ratio showed no overlap between Dahuangli and any reference at the individual-fruit level in this dataset, while firmness alone leaves a marginal overlap zone, motivating the two-proxy first tier. (**C**) Proposed two-stage authentication protocol: Stage 1 screening by the two proxies, followed by Stage 2 LC-MS/MS confirmation of flagged samples against the 32 Tier I biomarkers, with the 104 Tier II metabolites available for extended pattern matching and routine quality control.

## Data Availability

The original contributions presented in this study are included in the article and Supplementary Materials. Further inquiries can be directed to the corresponding authors.

## Supplementary Materials

The following supporting information can be downloaded at: https://www.mdpi.com/article/10.3390/foods15193457/s1, Figure S1: Pearson correlations among the four QC injections and the distribution of peak-area coefficients of variation; Figure S2: OPLS-DA score plots for the four pairwise comparisons of Dahuangli with each reference variety; Figure S3: permutation test results (200 permutations) validating the four OPLS-DA models; Figure S4: nine-panel comparison of the eight measured quality traits plus the derived sugar–acid ratio across the five varieties; Table S1: the 136 core Dahuangli-enriched metabolites, with chemical class, VIP values, fold changes, and BH-adjusted *p*-values in each comparison; Table S2: the 32 Tier I candidate biomarkers, with chemical class, minimum fold change, BH-adjusted *p*-values, per-variety mean ± SD, and within-variety CV; Table S3: quality-trait measurements for all varieties and replicates; Table S4: MRM annotation parameters (Q1/Q3 transitions, ionization mode, identification confidence level, CAS) for the 32 Tier I candidate biomarkers; Table S5: complete dataset of all 2335 detected metabolite features, with annotation, peak-area values for all samples and QC replicates, and BH-adjusted *p*-values for the four comparisons; Table S6: PCA explained-variance table and the top-25 contributing features for PC1 and PC2.

## Abbreviations

The following abbreviations are used in this manuscript:

GI	Geographical Indication
WTM	Widely Targeted Metabolomics
UHPLC-MS/MS	Ultra-High Performance Liquid Chromatography-Tandem Mass Spectrometry
QC	Quality Control
CV	Coefficient of Variation
PCA	Principal Component Analysis
OPLS-DA	Orthogonal Partial Least Squares-Discriminant Analysis
VIP	Variable Importance in Projection
SDM	Significantly Differential Metabolite
MRM	Multiple Reaction Monitoring
FC	Fold Change
min FC	Minimum Fold Change
HSD	Honestly Significant Difference
ANOVA	Analysis of Variance
ESI	Electrospray Ionization
PTFE	Polytetrafluoroethylene
MWDB	MetWare Database
NMR	Nuclear Magnetic Resonance
GC-O	Gas Chromatography-Olfactometry
TCA	Tricarboxylic Acid
δ^13^C	Carbon-13 stable isotope ratio
δ^15^N	Nitrogen-15 stable isotope ratio
δD	Deuterium stable isotope ratio
