# Supplementary material for: Candidate Metabolomic Biomarkers of the Geographical-Indication Pear Gaoping Dahuangli (Pyrus bretschneideri Rehd.): An Exploratory Widely Targeted Metabolomics Study
Source: Foods. 2026 Sep 27;15(19):3457. doi: 10.3390/foods15193457 (PMC13664191; doi:10.3390/foods15193457)
Supplement: Supplementary file 1 [file foods-15-03457-s001.zip › Supplementary Materials Figure S1-S4.pdf]

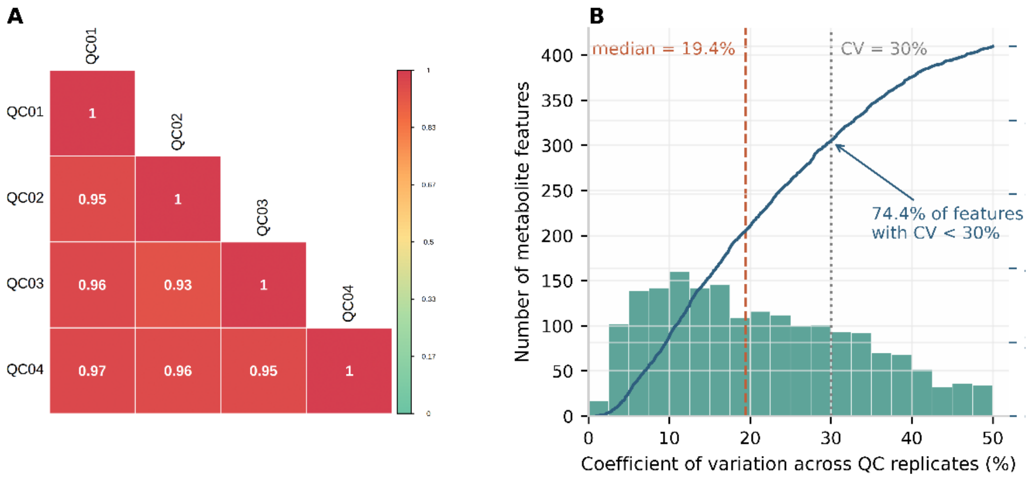

Figure S1: Pearson correlations among the four QC injections and the distribution of peak-area coefficients of variation. (**A**) Pearson correlations among the four quality-control (QC) injections (r = 0.93–0.97). (**B**) Distribution of peak-area coefficients of variation (CV) across QC replicates for all quantified features; the dashed line marks the median CV (19.4%) and the dotted line the 30% threshold. The blue cumulative curve (right axis) shows that 74.4% of features have CV < 30%.

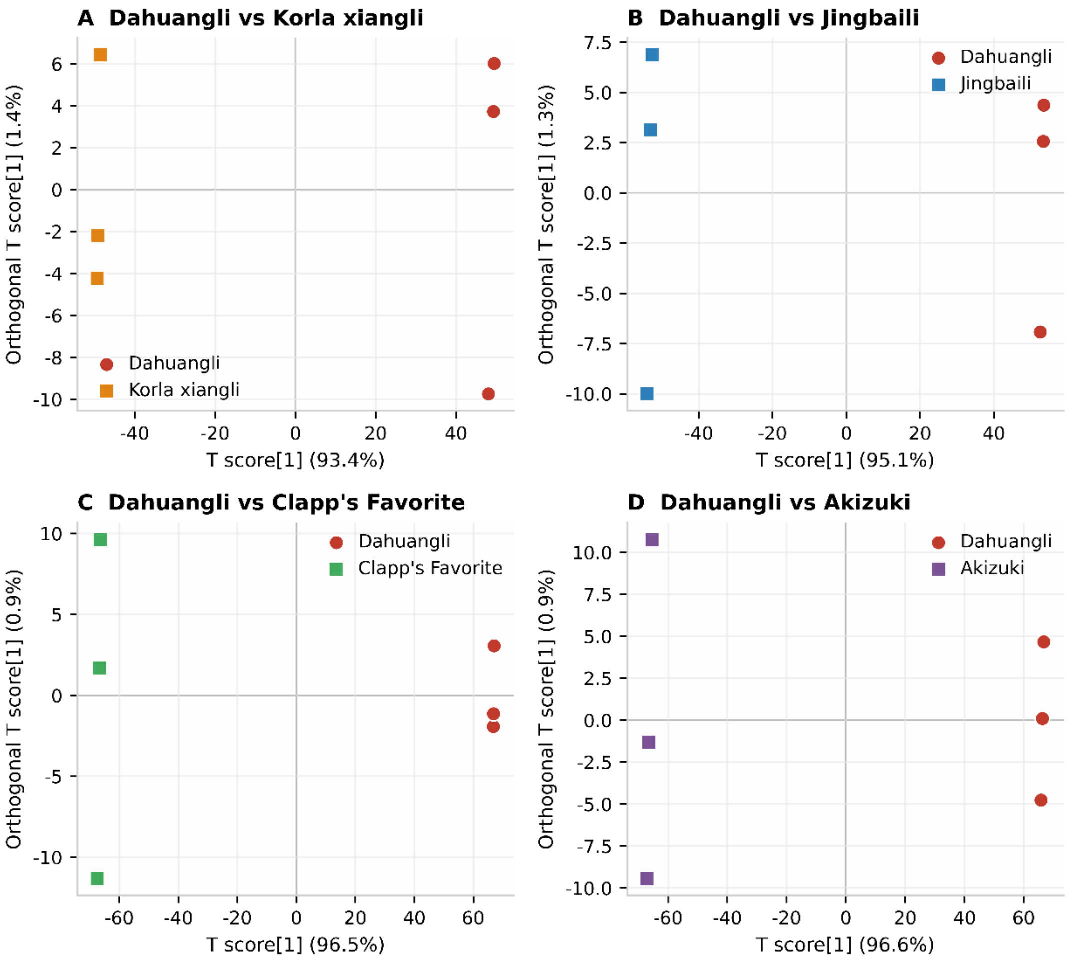

Figure S2: OPLS-DA score plots for the four pairwise comparisons of Dahuangli with each reference variety. (A) Dahuangli vs Korla xiangli; (B) Dahuangli vs Jingbaili; (C) Dahuangli vs Clapp's Favorite; (D) Dahuangli vs Akizuki. Dahuangli replicates (circles) separate completely from each reference cultivar (squares) along the predictive component in all four models.

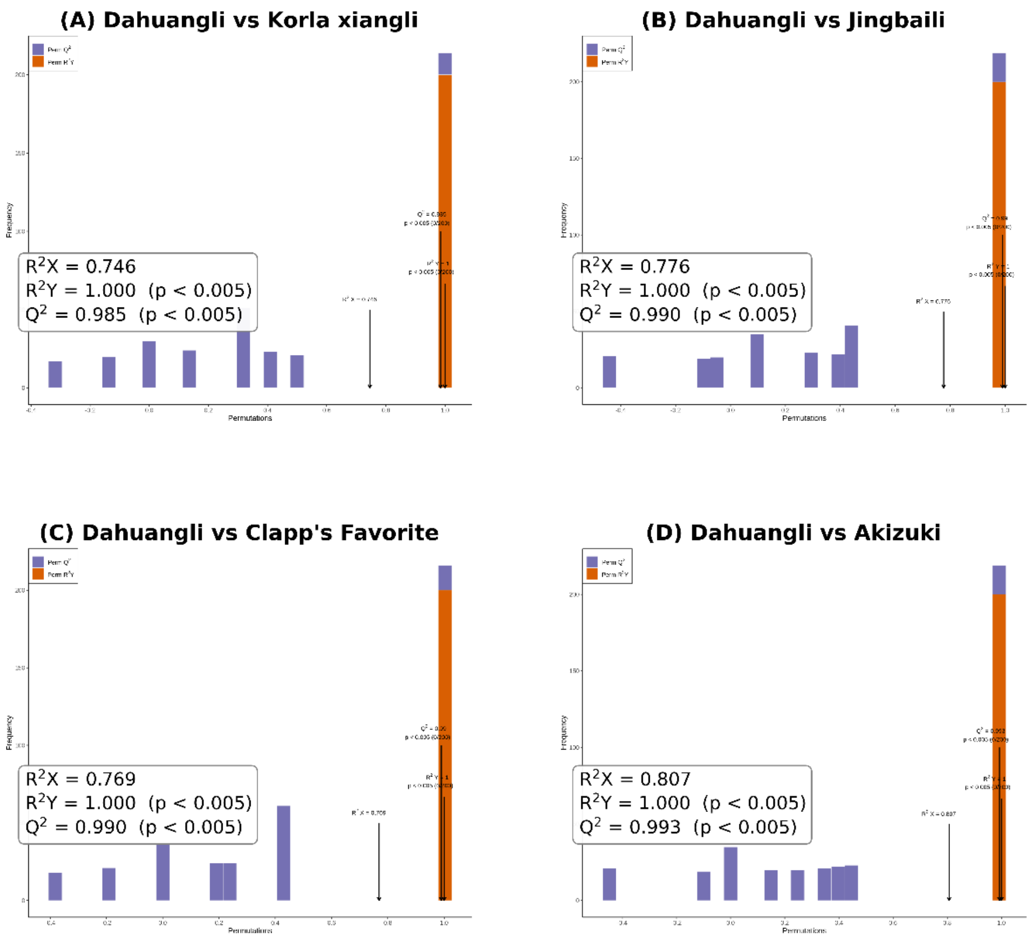

Figure S3: permutation test results (200 permutations) validating the four OPLS-DA models. (A) Dahuangli vs Korla xiangli; (B) Dahuangli vs Jingbaili; (C) Dahuangli vs Clapp's Favorite; (D) Dahuangli vs Akizuki. Histograms show the frequency distribution of permuted R^2Y (orange) and Q^2 (purple) values; the original model statistics (annotated in each panel, $R^2Y = 1.000$, $Q^2 = 0.985–0.993$) far exceed all permuted values ($p < 0.005$, 0/200), indicating no overfitting.

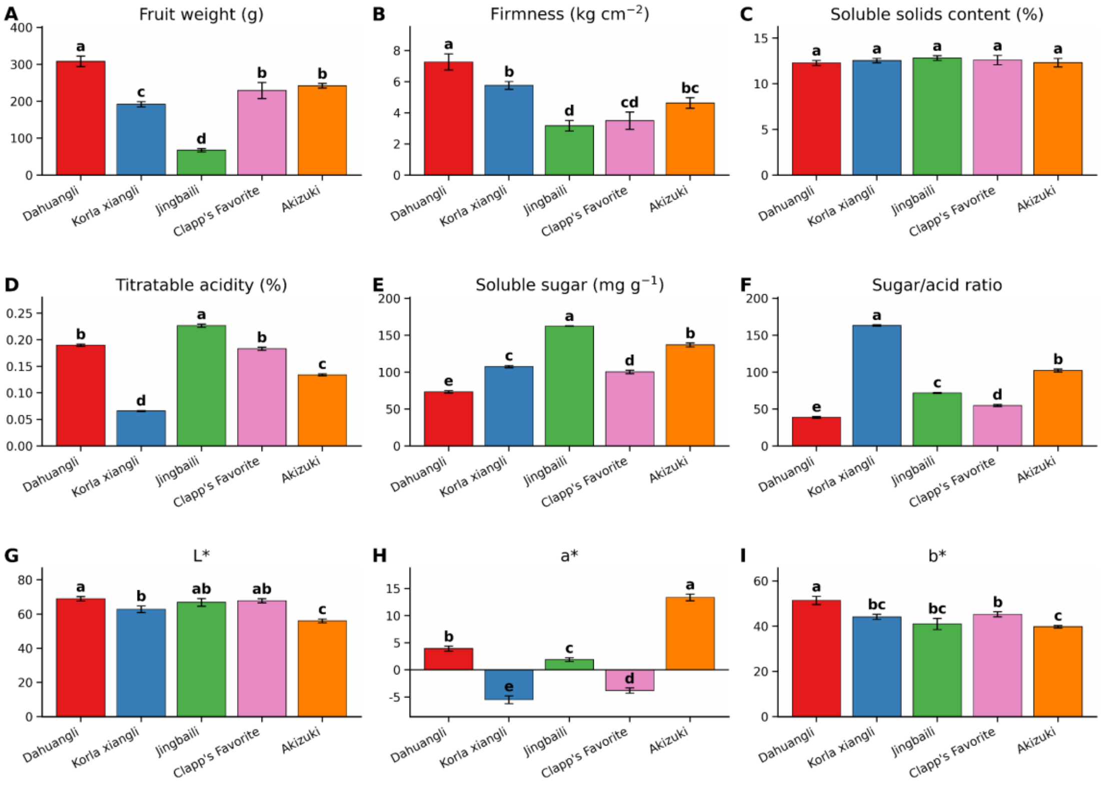

Figure S4: the eight quality traits across the five varieties. (A) Single-fruit weight; (B) firmness; (C) soluble solids content; (D) titratable acidity; (E) soluble sugar; (F) sugar–acid ratio; (G–I) skin colour coordinates L* (lightness), a* (redness) and b* (yellowness). Bars show the mean ± SE; different lowercase letters above bars indicate significant differences among cultivars.
